# An Algorithm for Soft Sensor Development for a Class of Processes with Distinct Operating Conditions

**DOI:** 10.3390/s24061948

**Published:** 2024-03-19

**Authors:** Darko Stanišić, Luka Mejić, Bojan Jorgovanović, Vojin Ilić, Nikola Jorgovanović

**Affiliations:** Faculty of Technical Sciences, University of Novi Sad, 21000 Novi Sad, Serbia; darkos@uns.ac.rs (D.S.); bojan.jorgovanovic@uns.ac.rs (B.J.); vojin@uns.ac.rs (V.I.); nikolaj@uns.ac.rs (N.J.)

**Keywords:** soft sensor, neural networks, distinct operating conditions, industrial application, cement fineness

## Abstract

Soft sensors are increasingly being used to provide important information about production processes that is otherwise only available through off-line laboratory analysis. However, usually, they are developed for a specific application, for which thorough process analysis is performed to provide information for the appropriate selection of model type and model structure. Wide industrial application of soft sensors, however, requires a method for soft sensor development that has a high level of automatism and is applicable to a significant number of industrial processes. A class of processes that is very common in the industry are processes with distinct operating conditions. In this paper, an algorithm that is suitable for the development of soft sensors for this class of processes is presented. The algorithm possesses a high level of automatism, as it requires minimal user engagement regarding the structure of the model, which makes it suitable for implementation as a customary industrial solution. The algorithm is based on a radial basis function artificial neural network, and it enables the automatic selection of the model structure and the determination of model parameters, only based on the training data set. The testing of the presented algorithm is done on the cement production process, since it represents a process with distinct operating conditions. The results of the test show that, besides providing a high level of automatism in model development, the presented algorithm generates a soft sensor with high estimation performance.

## 1. Introduction

In the process industry, much of essential process information, which mainly refers to product quality or production process performance, can be obtained only from off-line laboratory analysis. These laboratory analyses are performed in relatively large time intervals, usually every hour or every few hours, which is why values obtained from the laboratory are not suitable for direct use in process control and why deterioration in the production process performance is sometimes detected too late. There are two opposite situations that can be characterized as deterioration in the production process performance: production of the product with quality below the target limits, and production of the product with quality exceeding the target limits. While the first situation clearly presents the problem in production, because the product must be discarded or might require additional treatment, the second situation, although it results in a product that can be brought to market, is also a very significant problem because the product is produced with excess energy consumption and an increased carbon footprint. It is clear that real-time product quality control is essential for keeping products within the target limits; eventually, with stable and reliable control performance, even providing conditions for narrowing the target limits to decrease the carbon footprint. Since in a significant number of production processes, a real-time product quality control approach is not possible to implement based on laboratory analysis, there is a need to provide the required process information in an alternative way. The solution that overcomes this problem is based on the development of a model that uses signals measured directly in the process for on-line estimation of the information, which is usually obtained in the laboratory. Estimated values that are obtained in this way can be used directly in control loops. The models developed for these types of applications are called soft sensors. There are different approaches to soft sensor development, but the complexity of the production process and uncertainties in determining the connection between laboratory values and signals that are measured in the process are reasons why soft sensors in the process industry are mainly based on black box or gray box models. The black box approach is successfully applied to different processes, from the cement industry [1,2,3,4] and chemical processes [5,6,7] to water treatment [8,9,10], energy production [11] and the oil industry [12,13,14]. Since it proved appropriate for the development of soft sensors for a variety of industrial processes, the black box approach has the potential to be used as a basis for a wide industrial implementation of soft sensors.

Different model types have been used for the development of black box models for soft sensors in industrial processes, from models based on different types of Neural Networks [1,3,6,8,9,10,11,14], which represent the most common class of models, to Support Vector Regression [2,9], Neuro Fuzzy [13], Fuzzy Modeling [15], Linear Regression [16], and various other types of models. Determining the model type that is suitable for developing soft sensors for all industrial processes is, of course, not possible. However, using soft sensors as a customary solution in an industry application requires developing approaches that can be used relatively easily for building the models for a certain class of processes that are common in the process industry. One class of processes, which is very significant for the process industry, is the one in which there is the production of multiple product types. Typical examples of this are the grinding of the final product in mills in cement or lime plants. The other class of processes, which is somewhat similar to the first one, are production phases in which different types of raw materials can be used during production, resulting in different operating conditions. For this, typical examples are clinker production in cement kilns or lime production in lime kilns, which can use ore extracted from different locations and with different characteristics, or fuels of different quality. It is obvious that these two classes of industrial processes have distinct operating conditions under which they operate. When developing soft sensors for this class of processes, the model of the process, because of different product types or raw material characteristics, must depict process behavior for several different operating conditions. If the measured values from the process, which present potential model inputs, are observed, they are usually grouped in the input space, forming groups that depict different operating conditions. In the case study that is included in this paper, the grouping of the measured values in the input space will be demonstrated for the process in the cement industry. However, a similar situation can be observed in various other processes, from the chiller system, which is analyzed in [17], to the electric arc furnace, for which in, [15], data were grouped in four distinct clusters during soft sensor development, and the oil refinery’s distillation column, for which, in [12], five subsets of data depicting different working conditions were identified.

The characteristics of industrial processes with distinct operating conditions indicate that the approach based on a radial basis function artificial neural network (RBF ANN) should be suitable for soft sensor development in this class of processes. Placing the centers of radial basis functions in the parts of input space in which inputs are grouped for different operating conditions should provide the basis for the development of the appropriate process model. To develop an approach that is suitable to be implemented as a customary industrial solution, besides choosing the appropriate model type for this class of processes, it is important to provide a procedure for model development that is straightforward and requires minimal user engagement. This is exactly what is presented in this paper, a generic algorithm for creating a soft sensor without the need for users to contribute to the structure of the underlying model.

**Figure 1:**
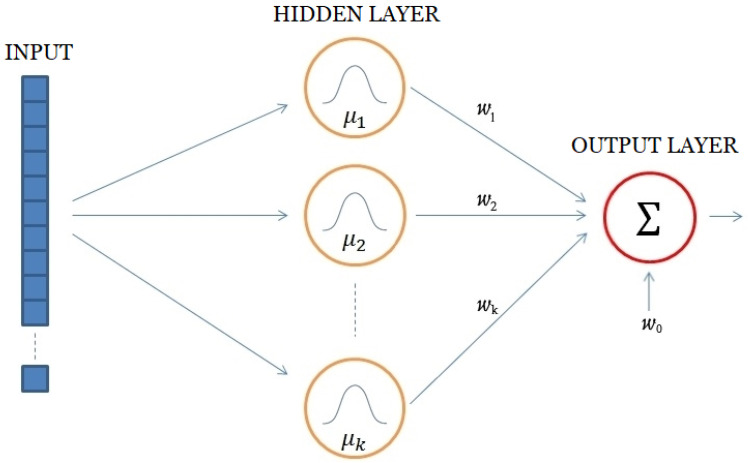
RBF ANN architecture.

The basic architecture of RBF ANN always consists of three layers. This type of neural network is always based on the input layer (input), the hidden nonlinear layer and the output layer, as shown in Figure 1 below.

The input in this type of ANN is viewed as a single point in multidimensional space, where the number of dimensions is defined by the number of signals based on whose values the regression is performed. The specific position of that point in multidimensional space is determined by the values of each of the signals that are chosen to be inputs in this artificial neural network.

The hidden layer consists of k neurons that contain radially based functions. Each of these neurons describes a part of the previously mentioned multidimensional space. In each hidden neuron, there is a radial basis function, usually a Gaussian function defined by center and width. The output of this function depends on the distance between the center of the function and the point defined by the values of the input to the artificial neural network. One way of describing the activation function of neurons in the hidden layer, which was used in this paper, is shown in (1):

(1) $$\phi_{i}\left(x\right)=e^{-\frac{{\left|\left|x-c_{i}\right|\right|}^{2}}{2\sigma_{i}^{2}}},$$

where $c_{i}$ represents the center of the Gaussian function of the i-th neuron of the hidden layer, and $\sigma_{i}$ is the width of it.

The output layer of this type of neural networks always has a linear activation function (2):

(2) $$y=\sum_{i=1}^{k}\omega_{i}{\cdot \phi }_{i}+\omega_{0},$$

where $\phi_{i}$ represents the output of the i-th neuron of the hidden layer.

There are various algorithms for training RBF ANN models, but they are mostly based on a trial-and-error approach and require significant user engagement for adjusting the model structure by evaluating training results to obtain a model that has adequate performance; therefore, they are not suitable for the implementation of a customary industrial solution. Training RBF ANN requires configuring radial basis functions in the hidden layer and determining weights that connect the hidden layer and the output layer. Configuring the hidden layer includes determining the number of centers, selecting the centers, and determining the parameters of the radial basis functions. The method based on orthogonal least squares is presented in [18] which selects the RBF centers from the training set and determines the number of the centers, while weights are calculated by using the least squares (LS) algorithm with the fixed parameters of RBF. In [19], this method is used for the development of a model for ore grade estimation in an offshore placer gold deposit. A similar approach, in which RBF parameters are fixed and weights are calculated with the LS algorithm, was presented in [20], where the number of centers and selection of the centers from the training set is done by using the genetic algorithm. Another algorithm that calculates weights in the same way and is focused on determining the number of centers by using fuzzy logic is presented in [21]. It calculates the RBF parameters based on the distance from the nearby centers, without evaluating how the computed parameters influence the model performance. The number of centers in the algorithm that is presented in [22] is determined by using Stein’s unbiased risk estimator; positions of the centers are determined from the training set by using subtractive clustering [23]; weights are calculated based on the LS, and RBF parameters are kept constant. There are training algorithms that require a detailed theoretical understanding of the process, like the one presented in [24], which uses the first principles model during the training to evaluate RBF ANN model performance; this makes it unsuitable for implementing as a customary solution. Another training approach that requires significant theoretical insight about the process is presented in [25], where a hybrid modeling approach is presented in which prior knowledge about the process is included in the model structure. The training algorithm presented in [26] can be used to determine all RBF ANN parameters except the number of centers, but a stochastic approach with on-line learning that uses a gradient decent algorithm to determine centers can have results in which the initial placement of the centers and organization of the training data can significantly influence the placing of the centers. This does not guarantee that centers will be placed based on the grouping of the data in the clusters, which define distinct operating conditions; this was one of the main motivations for choosing RBF ANN for modeling.

As it can be seen, there are some methods for RBF ANN model training that require decreased user engagement, but they still lack the level of automatism needed for industry applications, or they determine only some of the model parameters, while the remaining model parameters are kept fixed or determined in a simplified way. Also, some of the training methods are developed for specific applications and those methods are not directly transferable to the soft sensor implementation problem. For the implementation of a customary solution that can be used to develop soft sensors for industrial processes with distinct operating conditions, a new comprehensive algorithm should be specified, one that can be used to configure all RBF ANN parameters with a high level of automatism. In this paper, an algorithm is presented in which the RBF ANN model is used for the development of soft sensors for industrial processes with distinct operating conditions. The algorithm presents automatic procedures for model structure selection and model training, and it can provide a basis for the customary solution for industrial applications in which soft sensors are developed for these types of processes.

This paper is organized as follows: in Section 2, the proposed algorithm for soft sensor development based on RBF ANN is presented and described in detail. A case study from the cement industry is presented in Section 3 to demonstrate the applicability of the proposed algorithm to industrial processes with distinct operating conditions. Section 3 consists of a description of the process used in the case study, an analysis of the process, and a presentation of the performance of the developed soft sensor. Conclusion and discussion are given in Section 4.

## 2. Methodology Description

The hybrid training algorithm for RBF ANN presented here provides procedures for determining all parameters of the model, from the number and positioning of the centers to the values of RBF parameters and output weights. None of the model parameters are kept fixed or computed from simple expressions; all parameters are determined by procedures that are based on criteria aimed at improving model structure or model performance. It also includes procedures for parameter initialization, so that the resulting model does not depend on the initial guess of the parameter values, but only on the points from the training data. Industrial implementation of this approach for soft sensor development should provide a high level of automatism and minimal user engagement. In this training algorithm, the center selection is done by clustering data using the subtractive clustering and k-means method, while the number of centers is determined based on the Akaike information criterion (AIC) [26]. Output weights are determined by propagating in the forward direction and using the least squares algorithm, while the optimal widths of radially based functions are determined by backward propagation and the gradient method.

### 2.1. Selection of Centers

The selection of centers is based on the clustering methods, which ensure that centers are placed in the input space in such a way that the areas of input space with a higher density of data points in the training data set correspond to a higher number of centers, which are evenly distributed. These areas with the higher density of data points match the areas in which the majority of the system behavior is exhibited, which also reflect operating conditions in which the system mainly operates. Placing the centers in such a way enables the radial basis functions, which are determined by those centers, to describe the system operation more precisely in the parts of the input space which are of main interest for the model. To eliminate the possibility that the resulting model depends on the initial guesses of the centers of radial basis functions, training data are first clustered using subtractive clustering, as this method ensures consistency of the determined clusters for the same chosen parameters [22]. Furthermore, the position of the determined cluster centers is fine-tuned using the k-means method.

#### 2.1.1. Subtractive Clustering

The method of subtractive clustering assigns a certain potential to every sample of training data, based on which the cluster centers are found during the iterative procedure. Initially, the calculation of potential for each sample $x_{i}$, in the training data set, which contains N samples, is done. The potential based on the density of training data samples is calculated according to Equation (3):

(3) $$P\left(x_{i}\right)=\;\sum_{j=1}^{N}e^{\frac{-\left\|x_{i}-x_{j}\right\|}{\left(r_{a}^{2}/4\right)}}.$$

The parameter $r_{a}$ is the radius within which the surrounding points have a significant influence on the calculated potential. After the initial calculation of the potentials, an iterative procedure for determining cluster centers is performed, starting with the cluster l=1. In the first step, the sample with the highest potential is determined as the center $c_{l}$ of the cluster l. In the second step, the potential of each sample in the training data set is reduced by the influence factor of the already determined center, according to Equation (4):

(4) $$P\left(x_{i}\right)=P\left(x_{i}\right)-P(c_{l})\;e^{\frac{-\left\|x_{i}-c_{k}\right\|}{\left(r_{b}^{2}/4\right)}}.$$

This significantly decreases the potential of the points in the vicinity of the selected center, and the radius $r_{b}$ is usually selected to have a value of $1.5\;r_{a}$. In the third step, the stopping criterion is evaluated, and, if the criterion is not met, the procedure continues with the selection of the l=l+1 cluster center, from the first step. The stopping criterion used in this paper reaches the required number of centers. When the subtractive clustering method is used to also determine the number of clusters, the stopping criterion is based on the comparison of the highest potential of the remaining samples with the potential that was used to select the first cluster center $P(c_{1})$.

#### 2.1.2. k-Means Method

The k-means method is also an iterative algorithm. Initially, the desired number of clusters is chosen, as well as the initial centers of the clusters which are commonly chosen at random. After that, the iterative process can start. A single iteration of this method consists of only two steps: assignment step: each sample from the training data is assigned to the closest center of a cluster; update step: the center of each cluster is recalculated as the mean value of all assigned samples. The selection of cluster centers is finished when no change is detected between two consecutive iterations in the assignment step.

#### 2.1.3. Selection of Centers by Using Subtractive Clustering and k-Means

**Figure 2:**
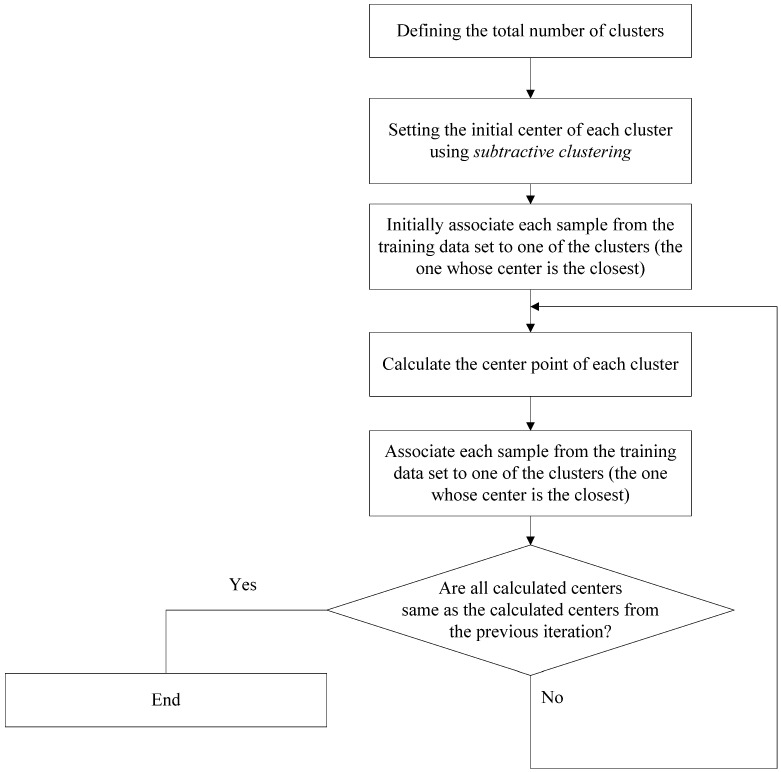
Selection of centers flow diagram.

The steps for the selection of centers by using these methods are shown in Figure 2.

### 2.2. Calculating the Widths of Radially Based Functions

The combination of the gradient method and the back-propagation method has found exceptional application in the training of artificial neural networks based on perceptrons (feed-forward artificial neural networks—FF ANN). This combination provides iterative procedure for parameter selection which minimizes the model error and is based on two basic steps that need to be applied to each observation from the training data set:

- Calculation of the estimated output based on the current network configuration and the input value in that observation, and, based on that, finding the error between the estimated and actual value of that observation;
- Correction of the configuration and parameters of the artificial neural network based on the calculated error and the gradient method;

For the implementation of the gradient method and the back-propagation method, it is necessary to define how the correction of the configuration of the artificial neural network will be performed. It is necessary to define the error function that will be minimized during the training algorithm, and then to determine the impact of changing each of the adjustable parameters of the artificial neural network on that function. The most commonly used error function that needs to be minimized is shown in Equation (5) as cost_fn, where N represents the number of observations in the training set, $y_{i}$ and $x_{i}$ the actual output value and the input value in the current observation, respectively, and $f\left(x_{i}\right)$ the estimated output value based on the current observation.

(5) $$cost\_fn=\;\sum_{i=1}^{N}\frac{{\left(y_{i}-\;f\left(x_{i}\right)\right)}^{2}}{2}$$

Using a combination of the gradient method and the back-propagation method, the widths of radially based functions in hidden neurons are adjusted. For this reason, it is necessary to find the influence of the change in the width of each radially based function on the change of the function whose minimization is targeted. Mathematically, this can be written as $\frac{dcost\_fn}{d\sigma_{j}}$ where $\sigma_{j}$ represents the width of the radial function of the j-th hidden neuron and can be determined in the following way (6):

(6) $$\frac{dcost\_fn}{d\sigma_{j}}=\;\frac{dcost\_fn}{df\left(x_{i}\right)}\cdot \frac{df\left(x_{i}\right)}{d\sigma_{j}}=\;\frac{dcost\_fn}{df\left(x_{i}\right)}\cdot \frac{df\left(x_{i}\right)}{d\phi_{j}\left(x_{i}\right)}\cdot \frac{d\phi_{j}\left(x_{i}\right)}{d\sigma_{j}}\;\;\;\;\;\;\;=-\left(y_{i}-f\left(x_{i}\right)\right)\cdot \omega_{j}\cdot \frac{d\left(e^{-\frac{{\left|\left|x_{i}-c_{j}\right|\right|}^{2}}{2\sigma_{j}^{2}}}\right)}{d\sigma_{j}}\;\;\;\;\;=-\left(y_{i}-f\left(x_{i}\right)\right)\cdot \omega_{j}\cdot e^{-\frac{{\left|\left|x_{i}-c_{j}\right|\right|}^{2}}{2\sigma_{j}^{2}}}\cdot \frac{4{\sigma_{j}\cdot \left|\left|x_{i}-c_{j}\right|\right|}^{2}}{4\sigma_{j}^{4}}\;\;\;\;=-\left(y_{i}-f\left(x_{i}\right)\right)\cdot \omega_{j}\cdot \phi_{j}\left(x_{i}\right)\cdot \frac{{\left|\left|x_{i}-c_{j}\right|\right|}^{2}}{\sigma_{j}^{3}}.$$

Once the effect of changing the widths of the radial functions on the cost function has been determined, it is possible to perform a width correction—training. It is standard that the training is done with a learning coefficient η whose value is in the range [0, 1] and determines the degree of correction for each width. The final expression for the change in the width of each individual radial function is shown in Equation (7).

(7) $$\Delta \sigma_{j}=\;\eta \cdot \frac{dcost\_fn}{d\sigma_{j}}$$

Initial widths of radial functions are set according to the spatial distribution of determined clusters. For all cluster centers, the same initial value is used, calculated with the expression $\sigma =\frac{d_{max}}{\sqrt{2N}}$ [11,27], which ensures that initial individual radial functions are not too peaked or too flat.

### 2.3. Calculating Output Weights

Output weights in RBF ANN are determined using the least squares algorithm. This algorithm finds the solution that maps the outputs of the radial basis functions to the targeted data with minimal residual error. The algorithm was proposed more than two centuries ago, and it selects model parameters which provide minimal sum of the squares of the differences between model output and actual value of the observed output for all data points in the training data set; that is, minimal error between model and data in the training set.

The calculation of the output weights using this method can be expressed as shown in Equation (8), where ω is the column vector of output weights, Φ is the matrix of outputs of the radial basis functions where rows represent observations and $y_{t}$ is the column vector of targeted outputs.

(8) $$\omega ={\left((\Phi^{T}\Phi \right)}^{-1}\Phi^{T})\cdot y_{t}$$

### 2.4. Determining the Number of Centers

Increasing model complexity provides reduction of the model error, but, at the same time, can result in overfitting. In order to select optimal model complexity, which is determined by the number of the radial basis functions, and prevent overfitting when incrementing the number of radial functions, the Akaike information criterion (AIC) was used. This criterion and its modified version have been proven successful in the determination of the best model by penalizing complexity in [28]. In each iteration, when model complexity is increased, in this case by adding one more radial basis function center, it evaluates how much of the error decrease is achieved. When the increase in the model complexity outweighs the reduction in the model error, the AIC stops model expansion. AIC can be computed with the following Equation (9):

(9) $$AIC=n\cdot \log\left({\dot{\sigma }}^{2}\right)+2\cdot K,\;{\dot{\sigma }}^{2}=\;\sum \frac{{\varepsilon_{i}}^{2}}{n},$$

where N is sample size, $\varepsilon_{i}$ is the estimated residual for a candidate model with a specific number of radial functions and K is the number of estimated parameters. As shown in [29], for a small sample size, modified AIC should be used. This modified criterion ${AIC}_{m}$ is shown in Equation (10).

(10) $${AIC}_{m}=AIC+\frac{2\cdot K\cdot (K+1)}{n-K-1}$$

As an increment of the number of radial functions is done in every outer iteration of the proposed algorithm, at the end of every iteration, ${AIC}_{m}$ is calculated. With the increased model quality, the ${AIC}_{m}$ parameter will decrease. The proposed algorithm will use this to finish the training process when, with the increment of the number of radial functions, the value of the calculated ${AIC}_{m}$ parameter increases compared to the value from the previous iteration.

### 2.5. Implementation of the Proposed Hybrid Training Algorithm

**Figure 3:**
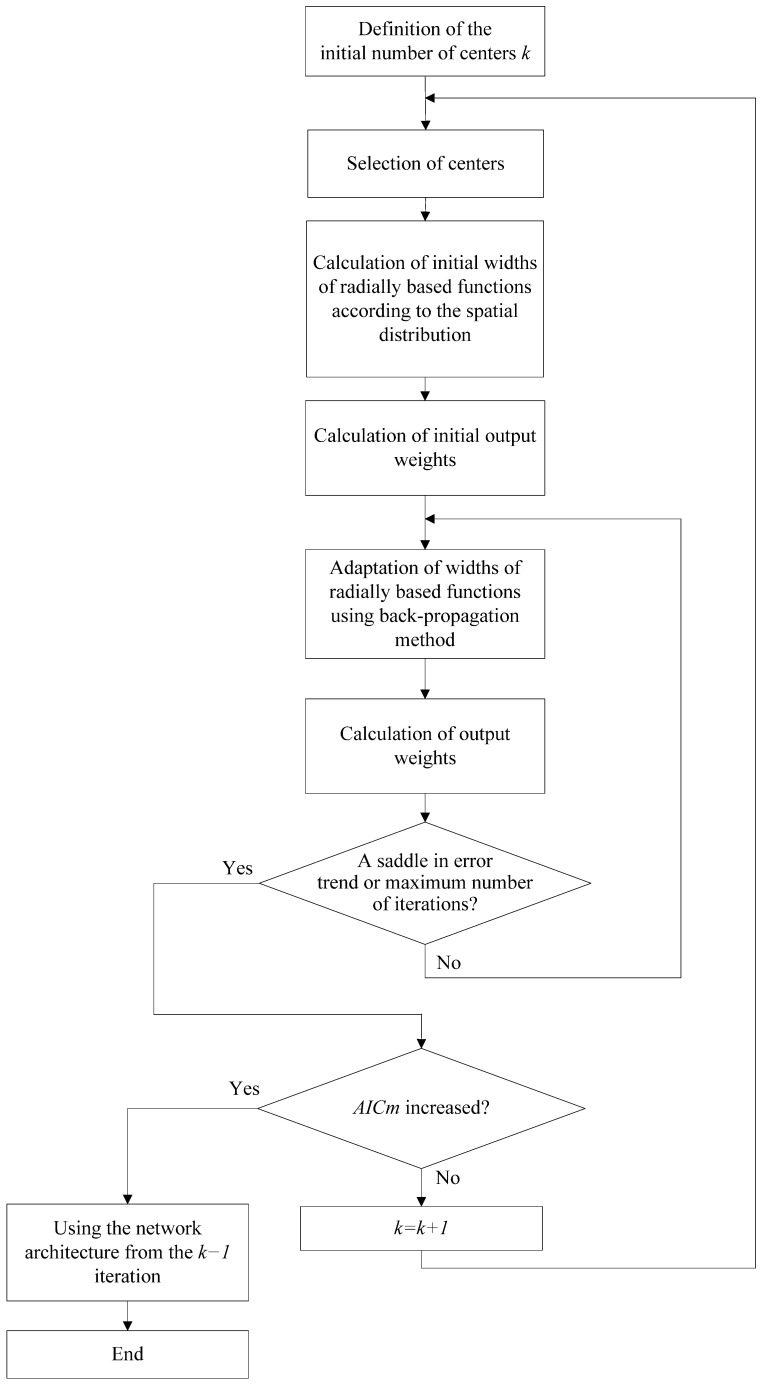
Flow diagram of the proposed hybrid training algorithm.

Based on the previous analysis, it is possible to combine all methods mentioned and implement a hybrid algorithm for training artificial neural networks with radially based functions. The implementation flow of the proposed hybrid training algorithm is shown in Figure 3.

## 3. Casy Study: Cement Fineness Estimation

### 3.1. Cement Grinding Process

**Figure 4:**
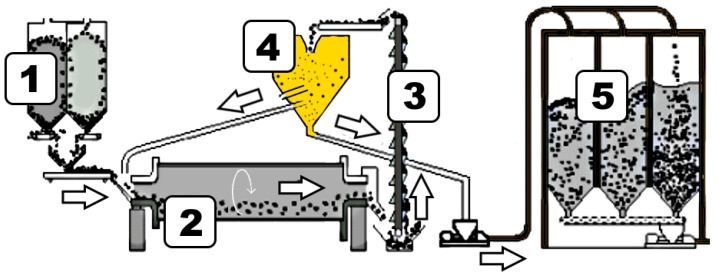
Cement grinding process.

In this paper, the proposed algorithm was used to develop a soft sensor for a cement grinding process. The results are then shown and compared with the already published results presented in [3]. This process was chosen given its distinct operating conditions, which are inherent due to the production of different types of cement on the output. Another reason why this specific process was chosen was the availability of the data and the overall feasibility with regard to the algorithm’s real-time efficacy evaluation. This process is visually presented in Figure 4.

The process of turning raw materials into fine dust in order to create cement, known as cement grinding, is the last step in the cement production process. The fineness of the finished product is one of cement’s most crucial characteristics. The primary component of Portland cement, the most popular kind of cement, is clinker, which is combined and processed in a mill with additional ingredients based on the particular recipe (Figure 4, marker 1). All materials are continuously moved from material silos to the ball mill (Figure 4, marker 2), since the grinding process is continuous. The material is then moved through multiple mill chambers, where steel balls are used to crush it while the mill rotates. After leaving the mill, the material is sent to a separator by means of a bucket elevator (Figure 4, marker 3). In the separator (Figure 4, marker 4), the part of the material that is finely ground is separated from the rest of the material as the final product. Finely ground material is then carried to the silos for storage of the finished product (Figure 4, marker 5). The material that, during the separator’s process of separation, did not pass as the final product, is rejected and returned to the mill input, where it is ground alongside the fresh material.

### 3.2. Data Set and Data Processing

The important part of soft sensor development is choosing measured values that are going to be used as model inputs. For continuous processes like cement grinding, this task comes with the additional requirement of determining the time delay between the measured value, which is chosen as the model input, and the process variable, which is estimated as the model output. The algorithm presented in this paper is focused on the model development of a soft sensor for which inputs are already selected, so the input selection can be done in any way that is suitable for the observed process. However, we find that the approach for input selection, as well as data preprocessing, presented in [30] is highly efficient for continuous processes, since it provides a way to choose signals and their time delays that contain the most information about the desired soft sensor output. It describes in detail how data for this type of industrial process should be preprocessed, and, after that, presents the automated algorithm that will select the inputs and their time delays, which should be used for a soft sensor. In this case, study inputs and their time delays were selected by using the approach from [30] which resulted in the set of inputs presented in Table 1.

**Figure 5:**
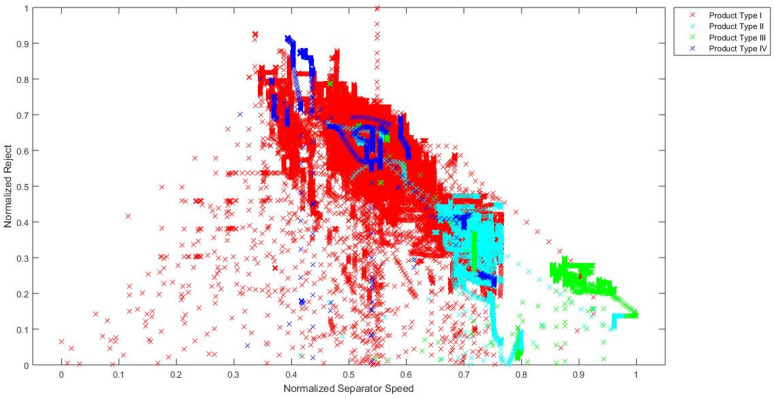
Normalized values of Rotational speed of separator and Mill reject through different product types.

**Figure 6:**
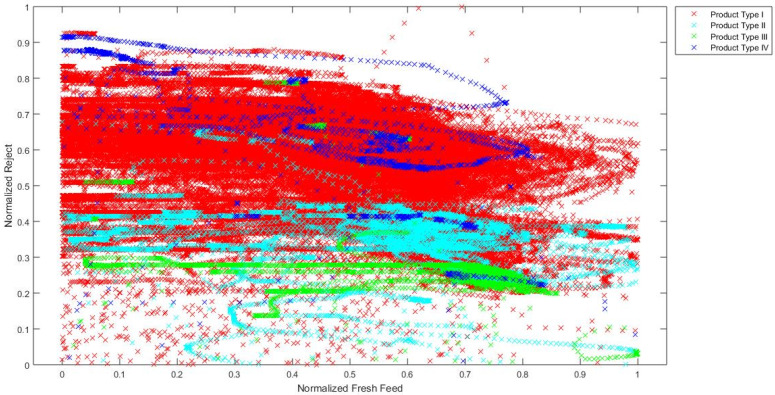
Normalized values of Mill fresh feed and Mill reject through different product types.

**Figure 7:**
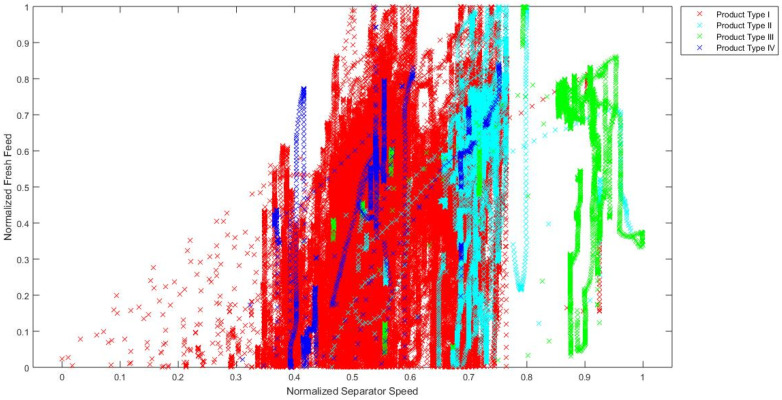
Normalized values of Rotational speed of separator and Mill fresh feed through different product types.

**Figure 8:**
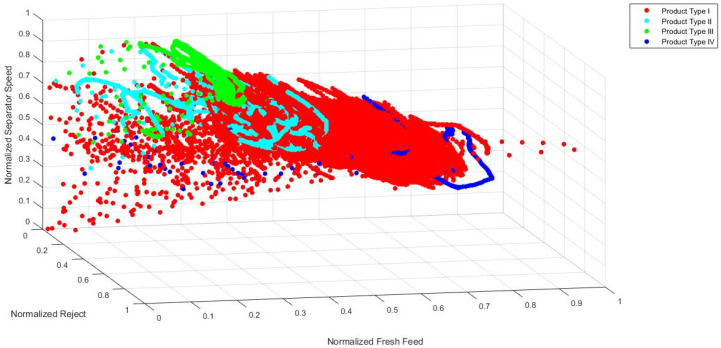
Normalized values of Mill fresh feed, Mill reject and Rotational speed of separator through different product types.

In order to confirm the hypothesis that the chosen industrial process is a process with distinct operating conditions, a visual analysis of signal values during the grinding of different types of cement is made. Although it is not possible to fully visually present how data are clustered in the 10-dimensional input space, a simple visual representation of data in the 2-dimensional and 3-dimensional input spaces gives a clear indication that input data are clustered in distinct areas for different product types. Training data are presented in 2D charts (Figure 5, Figure 6 and Figure 7) and subsequent 3D chart (Figure 8) for normalized values of 3 model inputs: Rotational speed of separator, Mill fresh feed and Mill reject. Different types of cement are presented in different colors. Figures show that, for a visual representation with two inputs, it looks like there is a grouping of the data based on the different product types, but with occasional overlapping of data from all product types. However, when using a visual representation with three inputs, it is clear that product types II, III and IV have significantly decreased overlapping of data, while there is still overlapping of all product types with product type I. As it can be seen from these figures, even when using only two or three inputs, the values of process variables are being grouped into different working regimes when different types of cement are ground, and the remaining inputs contribute to the grouping even more. Besides the grouping of data because of the different product types, the additional grouping is caused by the different properties of the material that is entering the mill. Since material is continuously flowing from the silos to the mill and there is no measurement of the properties of that material, it is not possible to clearly visualize and present how the changed material properties are influencing the grouping of the data. However, it is obvious from the process characteristics that this influence exists and the actual grouping of the data in the input space is even more significant than what can be seen based on the product types.

### 3.3. Results

It can be seen that the proposed algorithm ensures that, for the same data set, the same configuration of the soft sensor will be chosen no matter how many times the algorithm is run. In this section, results obtained from the data set related to the described cement grinding process are presented. The RBF ANN training data set consisted of 3600 samples, of which 70% were used for training and the other 30% were used for validation.

The soft sensor performance was examined in real-time on a real cement grinding process. The performance was quantified using the mean square error (MSE) and R-squared coefficient (R^2^) shown in Equations (11) and (12), respectively. In equations, ${ys}_{i}$ is the soft sensor estimation and ${yl}_{i}$ represents the laboratory obtained value in the industrial process.

(11) $$MSE=\sum_{i=1}^{n}\frac{{\left({ys}_{i}-\;{yl}_{i}\right)}^{2}}{n}$$

(12) $$R^{2}=1-\;\frac{\sum_{i=1}^{n}{\left({yl}_{i}-\;{ys}_{i}\right)}^{2}}{\sum_{i=1}^{n}{\left({yl}_{i}-\frac{1}{n}\;\sum_{i=1}^{n}{ys}_{i}\right)}^{2}}$$

**Figure 9:**
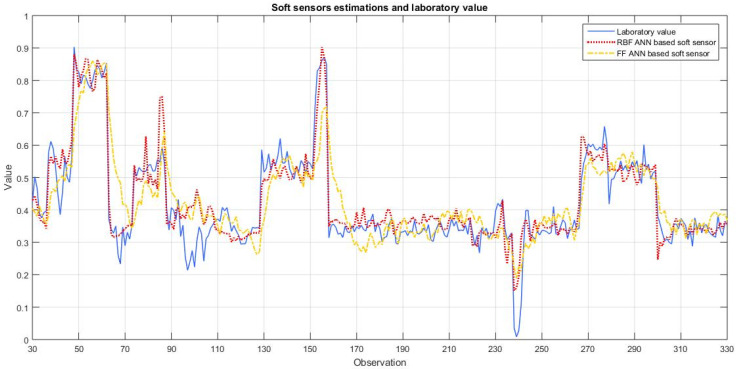
Normalized soft sensor estimations and laboratory value over time.

Since feed-forward artificial neural networks are the most widely used type of model for soft sensor development, the proposed soft sensor performance was compared to the FF ANN based soft sensor created according to [3]. The estimated values and laboratory values of cement fineness during online and real-time evaluation of both soft sensors can be seen in Figure 9. In Table 2, the mean square errors and R-squared coefficients for RBF ANN developed using the proposed algorithm and FF ANN proposed in [3] are presented.

It can be seen that the algorithm proposed in this paper not only provides a high level of automatism during model development, but also that the soft sensor developed using this algorithm achieves better results in the same industrial process compared to the soft sensor based on FF ANN.

## 4. Conclusions

This paper presents an algorithm for soft sensor development for a class of industrial processes with distinct operating conditions. The goal was to develop an algorithm that is suitable for this class of industrial processes and has a high level of automatism, which would enable it to be used as a customary solution for industrial applications. The algorithm specifies procedures for determining model structure and model parameters that provide optimal model performance based on the specified criteria. It is based on radial basis function artificial neural networks, subtractive and k-means clustering, least squares, gradient method, back-propagation and Akaike information criterion. A case study was made on a data set from regular cement production, as it was shown that cement production is a process with distinct operating conditions. A soft sensor for estimating the fineness of cement, which is the most important property of the cement grinding process, was developed. The performance of this soft sensor in real-time evaluation was shown and compared to the performance of a soft sensor based on the feed-forward artificial neural network, which is the most common type of model used in similar processes, and has already proven to have good performance in the estimation of cement fineness. The soft sensor that was developed using the proposed algorithm, besides providing automatic model structure selection, achieved better performance according to the mean square error and R-squared performance indexes. It is important to note that automatic simultaneous selection of the model structure and the determination of the model parameters in the presented algorithm requires more computational resources and increases the time needed for training models compared to training models for which structure is selected manually. However, this algorithm significantly decreases the overall time needed for model development, since manual selection of model structure, which is usually done by trying different models and comparing their performance, takes much more time, which is spent training multiple models with different model structures and analyzing their performance. The presented model performance and high level of automatism during model development indicate that the proposed algorithm is applicable for developing soft sensors in industrial processes with distinct operating conditions.

## Figures and Tables

**Table 1:** Inputs and their time delays, based on the selection from [30].

Signal Name	Time Delay [Minutes]
Mill fresh feed	19
Electrical ear chamber 2	19
Mill reject	25
Pressure after mill	14
Pressure before mill	28
Mill reject	1
Rotational speed of separator	0
Electrical ear chamber 2	1
Electrical ear chamber 1	18
Temperature of final product	2

**Table 2:** Comparison of mean square errors and R-squared coefficients between soft sensors.

Soft Sensor	MSE	R^2^
RBF ANN (proposed by this paper)	0.0103	0.7953
FF ANN (proposed in [3])	0.0167	0.4725

## Data Availability

Data used in this paper will be available on request.
